# Surgical Management of Right Atrial Mass Associated with a Vascular Access Catheter

**DOI:** 10.1155/2020/4590147

**Published:** 2020-06-10

**Authors:** David Ferreira, Anthony Le, John Khoo, Paul Nguyen, Manish Jain, Timothy Spicer, Craig Juergens

**Affiliations:** ^1^Department of Cardiology, Liverpool Hospital, NSW, 2170 Sydney, Australia; ^2^Department of Medicine, University of New South Wales, NSW, 2052 Sydney, Australia; ^3^Department of Cardiothoracic Surgery, Liverpool Hospital, NSW, 2170 Sydney, Australia; ^4^Department of Nephrology, Liverpool Hospital, NSW, 2170 Sydney, Australia

## Abstract

Atrial masses are an uncommon but serious clinical problem. The authors report a case of an atrial mass associated with a tunnelled vascular access catheter in an immunosuppressed haemodialysis patient. In the setting of immunosuppression with fevers, a broad differential for the atrial mass was considered. Multidisciplinary team review was pursued to guide management decisions. Ultimately, surgical excision of the mass was pursued with an excellent result. The causes and management of this complex clinical scenario are discussed.

## 1. Introduction

Atrial masses pose an infrequent diagnostic and management dilemma. The differential diagnoses are broad and include primary and secondary malignancy, benign neoplasms, thrombi, and infective foci [1, 2]. Assessment and management require integration of clinical, laboratory, and imaging investigations and may necessitate involvement from multiple specialty teams. In this report, the authors describe a case of a right atrial mass complicating immunosuppression for lupus nephritis and haemodialysis via a tunnelled vascular access catheter. Ultimately, a diagnosis of atrial thrombus was confirmed on histology. The considered differential diagnoses and management strategy will be described. The management of atrial thrombus associated with vascular access catheters will then be discussed.

## 2. Case Presentation

An 18-year-old female with lupus nephritis, treated with tacrolimus and rituximab, was referred for admission to hospital. She had, until recently, required haemodialysis via a tunnelled permanent vascular access catheter which was still in situ. Complicating her immunosuppression, *Cryptococcus neoformans* meningitis was diagnosed through lumbar puncture seven months earlier. She was initially treated with intravenous amphotericin and flucytosine and was subsequently placed on therapeutic oral fluconazole.

**Figure 1:**
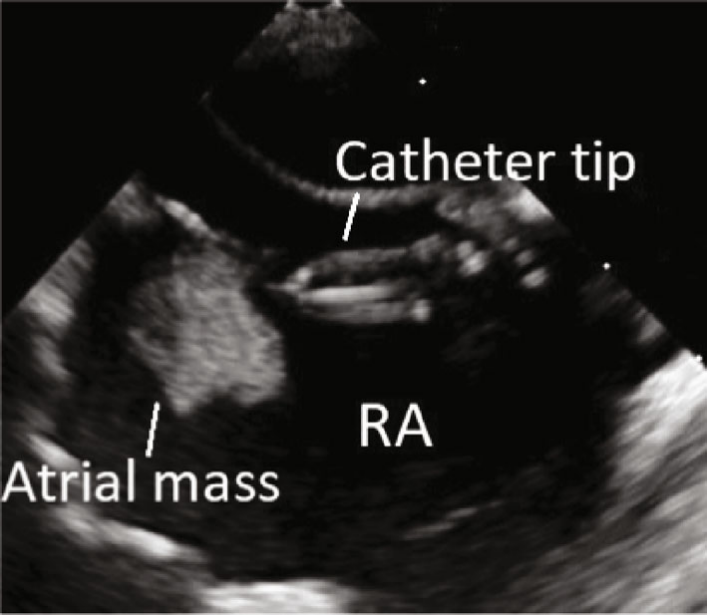
Transoesophageal echocardiography demonstrating vascular catheter tip abutting a right atrial mass.

Outpatient transthoracic echocardiography demonstrated a large echogenic mass in the right atrium at the tip of the vascular access catheter. Transoesophageal echocardiography confirmed an echogenic mass in the right atrium measuring 23 mm × 26 mm (see Figure 1) and a patent foramen ovale (PFO) with left to right shunt (see Clip 1). The differential diagnoses considered were that of atrial thrombus complicating vascular access, as well as an infective focus. A primary tumour was unlikely given a normal transthoracic echocardiogram four months before.

Inflammatory markers and septic screening with blood cultures were performed. C-reactive protein was 0.9 mg/L, and the neutrophil count was 9.5 × 10^9^. Three blood cultures were negative for bloodstream infection. Ventilation perfusion scanning revealed segmental pulmonary emboli. After multidisciplinary discussion, standard percutaneous catheter removal was deemed high risk due to potential embolisation. Anticoagulation with Apixaban was initiated for suspected atrial thrombus, and serial cardiac imaging was organised. Removal of the vascular access catheter was to be planned after thrombus resolution, and the patient was discharged [3].

Four weeks later, she represented with lethargy and fevers to 39.4 degrees Celsius. Inflammatory markers were elevated with a C-reactive protein of 157 mg/L and neutrophils of 10.2 × 10^9^. A repeat infective panel was performed, and empiric therapy with intravenous gentamicin and vancomycin was provided due to skin colonisation with methicillin-resistant *Staphylococcus aureus*. Blood cultures, including vascular catheter cultures, were positive for *Stenotrophomonas maltophilia*, and a diagnosis of line-associated bacteraemia was made. Infectious diseases input was sought, and broad-spectrum antimicrobials with intravenous trimethoprim-sulfamethoxazole and cefepime were administered. Regular therapeutic oral fluconazole was continued. Rapid clinical response to antimicrobial therapy was seen with defervescence within 24 hours.

Repeat transthoracic echocardiogram showed no interval change in the right atrial mass despite anticoagulation. This placed the diagnosis of thrombus in doubt, and in the setting of bacteraemia, complicating infection could not be excluded. After multidisciplinary review by infectious diseases, cardiology, cardiothoracic surgery, nephrology, and interventional radiology, definitive management of the atrial mass was deemed appropriate. This decision was made considering the diagnosis of thrombus was in doubt after anticoagulation failed to reduce its size, and in the setting of bacteraemia, complicating infection could not be excluded. Endovascular removal and thrombolysis were deemed high risk due to potential for embolisation. The decision was made to pursue open surgical thrombectomy, PFO closure, and vascular catheter removal. Brain magnetic resonance imaging (MRI) was negative for ischaemic stroke prior to on-pump cardiothoracic surgery.

**Figure 2:**
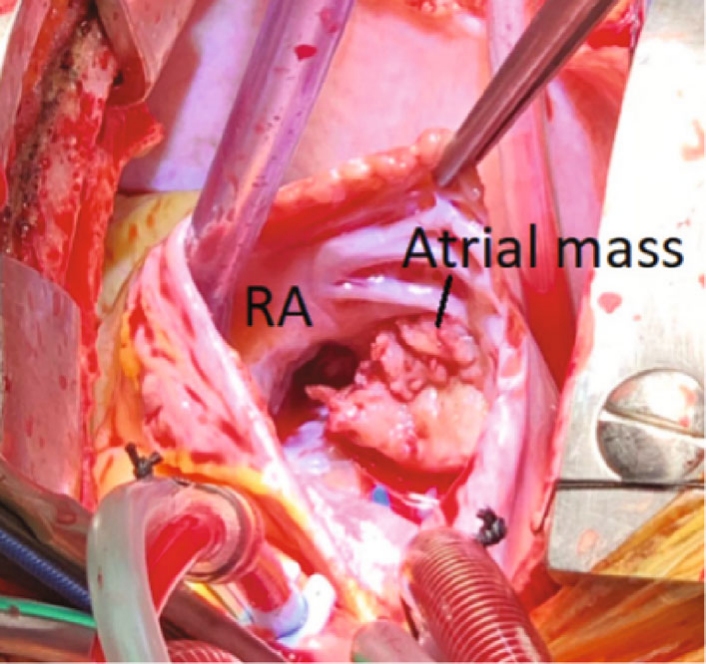
Intraoperative image of open right atrium with right atrial mass.

**Figure 3:**
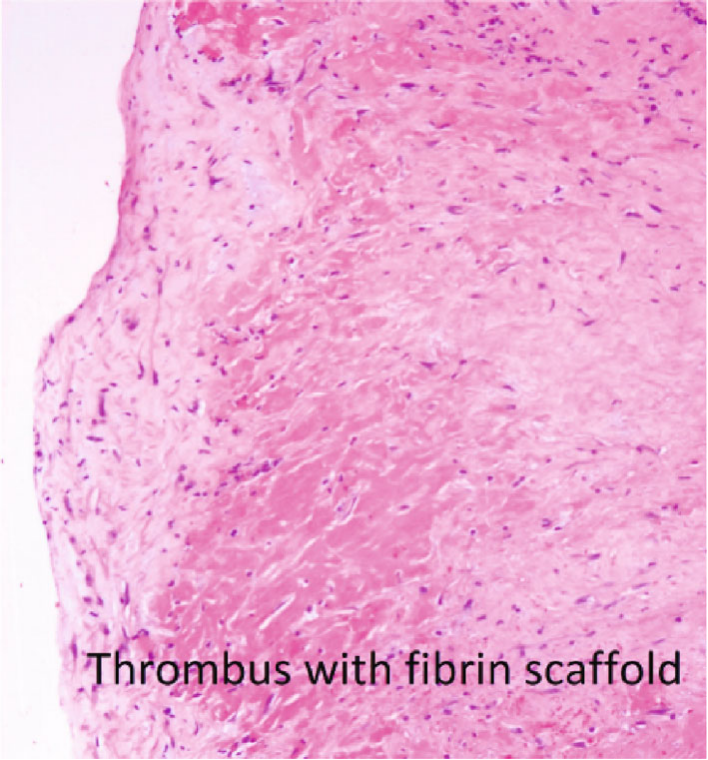
Histology of the atrial mass demonstrating organised thrombus with fibrin scaffold.

Surgery was performed (see Figure 2) with no postoperative complications, and she was discharged seven days later. Histology demonstrated organised thrombus with no evidence of abscess, granuloma, endocarditis, or myxoma (see Figure 3).

## 3. Discussion

Though infrequent, atrial masses can pose a significant diagnostic and management challenge. The differential diagnosis is broad and includes primary and secondary cardiac tumours, infective processes, and thrombi. Primary tumours are rare and have an incidence of 0.02% based on large autopsy series [4]. Secondary cardiac metastases occur 20 times more often, the most common primaries being mesothelioma, lung cancer, and melanoma [4]. Primary atrial mural endocarditis is rare with reported organisms including staphylococcus species, streptococci species, aspergillus, and candida [2, 5–7]. Secondary infection complicating myxoma has also been reported with similar bacterial pathogens [8].

In this case, the atrial mass was histologically confirmed as organised thrombus without an infective component. While atrial thrombi are rare, they can complicate 5-18% of vascular access catheters for haemodialysis patients [9, 10]. This is related to factors including the intra-atrial prothrombotic foreign body, frequent complicating infections in the immunosuppressed, and thrombotic predisposition in the chronic kidney disease population [11]. Atrial thrombi complicating vascular catheters are associated with mortality rates of 18.3-20.6%, most often due to pulmonary embolism, cardiac failure, and overwhelming sepsis [3, 12]. Mortality rates appear high (44%) in those managed without thrombus specific intervention [3].

Due to the infrequent nature of atrial thrombi complicating vascular catheters, there are no prospective data informing management. Prevention is better than cure, and in the haemodialysis population, fistula formation should be considered over vascular access catheters if feasible [12]. When a thrombus is present, removal of the vascular catheter is ideal [3]. Percutaneous removal may not be possible due to risk of thrombus dislodgement and life-threatening pulmonary embolism. Current retrospective reviews have shown that in 50% of cases, therapeutic anticoagulation was the initial treatment of choice. Primary surgical thrombectomy is pursued in 25% of patients while first line thrombolysis occurs in 7-17%. The choice of initial therapy is influenced by patient comorbidities, thrombus characteristics, and access to local surgical expertise. In those with contraindications to anticoagulation, thrombus size of greater than 6 cm or those with another indication for cardiothoracic surgery (e.g., infective endocarditis), a primary surgical strategy may be preferred [3, 12].

In patients chosen for anticoagulation, six months of therapy appears reasonable with serial imaging, close follow-up, and careful monitoring for infective, embolic, and cardiac sequelae [9]. A longer course of anticoagulation may be required if thrombus resolution is not achieved. Anticoagulation failure occurs in 30% of treated patients, and second-line options are thrombectomy or thrombolysis, though the latter is associated with failure rates of 30-75% [12].

When failing anticoagulation, particularly with the potential for life-threatening complications, surgical intervention for vascular access catheter associated atrial thrombus is a potential management option in complex patients [13, 14].

## Supplementary Materials

Supplementary Materials Clip 1: small patent foramen ovale with left to right shunt.

## References

[1] Mankad R., Herrmann J. (2017). Cardiac tumors: echo assessment. *Echo Research and Practice*.

[2] Soman S. O., Vijayaraghavan G., Padmaja N. P., Warrier A. R., Unni M. (2014). Aspergilloma of the heart. *Indian Heart Journal*.

[3] Stavroulopoulos A., Aresti V., Zounis C. (2012). Right atrial thrombi complicating haemodialysis catheters. A meta-analysis of reported cases and a proposal of a management algorithm. *Nephrology, Dialysis, Transplantation*.

[4] Silvestri F., Bussani R., Pavletic N., Mannone T. (1997). Metastases of the heart and pericardium. *Giornale Italiano di Cardiologia*.

[5] Hosokawa S., Okayama H., Hiasa G. (2018). Isolated left atrial infective mural endocarditis. *Internal Medicine*.

[6] Agrawal Y., Dada R., Dada J., Degregorio M. (2018). Primary mural infective endocarditis with associated central line infection. *BMJ Case Reports*.

[7] Chowdhury W., Lodhi M. U., Syed I. A., Rahim U., Miller M., Rahim M. (2018). Catheter-related Candida endocarditis on the right atrial septum - a case report. *Cureus*.

[8] Yuan S.-M. (2015). Infected cardiac myxoma: an updated review. *Brazilian Journal of Cardiovascular Surgery*.

[9] Shah A., Murray M., Nzerue C. (2018). Right atrial thrombi complicating use of central venous catheters in hemodialysis. *The Journal of Vascular Access*.

[10] Dilek M., Kaya C., Karatas A., Ozer I., Arik N., Gulel O. (2015). Catheter-related atrial thrombus: tip of the iceberg?. *Renal Failure*.

[11] Wattanakit K., Cushman M. (2009). Chronic kidney disease and venous thromboembolism: epidemiology and mechanisms. *Current Opinion in Pulmonary Medicine*.

[12] Tran M.-H., Wilcox T., Tran P. N. (2020). Cather-related right atrial thrombosis. *The Journal of Vascular Access*.

[13] Hussain N., Shattuck P. E., Senussi M. H. (2012). Large right atrial thrombus associated with central venous catheter requiring open heart surgery. *Case Reports in Medicine*.

[14] Akçay M., Deşer S. B., Gedikli Ö., Yüksel S., Gülel O. (2017). Successful management of complications after inappropriate positioning of a hemodialysis catheter. *Anatolian Journal of Cardiology*.

